# Toward the right treatment at the right time: Modeling the trajectory of cognitive decline to identify the earliest age of change in people with Alzheimer's disease

**DOI:** 10.1002/dad2.12563

**Published:** 2024-03-07

**Authors:** R. Asaad Baksh, André Strydom, Ben Carter, Isabelle Carriere, Karen Ritchie

**Affiliations:** ^1^ Department of Forensic and Neurodevelopmental Sciences, Institute of Psychiatry, Psychology, and Neuroscience King's College London Denmark Hill London UK; ^2^ The LonDownS Consortium Denmark Hill London UK; ^3^ South London and Maudsley NHS Foundation Trust Michael Rutter Centre London UK; ^4^ Department of Biostatistics and Health Informatics Institute of Psychiatry, Psychology and Neuroscience King's College London London UK; ^5^ INSERM, Institut de Neurosciences de Montpellier INM Montpellier France

## Abstract

**Introduction:**

Age is the greatest risk factor for Alzheimer's disease (AD). A limitation of randomized control trials in AD is a lack of specificity in the age ranges of participants who are enrolled in studies of disease‐modifying therapies. We aimed to apply Emax (i.e., maximum effect) modeling as a novel approach to identity ideal treatment windows.

**Methods:**

Emax curves were fitted to longitudinal cognitive data of 101 participants with AD and 1392 healthy controls. We included the Mini‐Mental State Examination (MMSE) and tests of verbal fluency and executive functioning.

**Results:**

In people with AD, the earliest decline in the MMSE could be detected in the 67–71 age band while verbal fluency declined from the 41–45 age band. In healthy controls, changes in cognition showed a later trajectory of decline.

**Discussion:**

Emax modeling could be used to design more efficient trials which has implications for randomized control trials targeting the earlier stages of AD.

## 1 INTRODUCTION

Dementia is the fifth leading cause of death worldwide^1^ and has a global prevalence which now exceeds 50 million people.^2^ Alzheimer's disease (AD) characterized by amyloid plaques, neurofibrillary tangles, and prominent impairments in memory abilities in the early stages is the most common cause of dementia.^3^ The global economic burden of AD and other dementia was an estimated $2.8 trillion in 2019 and is anticipated to increase to $4.7 trillion in 2030.^4^ These findings emphasize the need to increasingly direct research toward early intervention to reduce both the incidence and duration of illness.

There are currently many randomized controlled trials (RCTs) of disease‐modifying therapies (DMT) underway, and many more in the pipeline.^5^ These treatments seek to prevent or delay the development and progression of AD,^6^ but to date, their clinical efficacy has been poor.^7^ This limited success provides opportunities to consider novel approaches in RCT designs in critical aspects such as targeting participants at most risk for decline. By adding greater specificity to the recruitment age of prospective participants into studies, this may result in better identification of optimal intervention windows. The preclinical and prodromal phases of AD pathology can precede clinical onset by decades^8^ and early changes in cognition can be detected before a formal diagnosis.^9^, ^10^ There is growing evidence to suggest that the earlier the intervention, the higher the possibility of success.^11^ Intervening earlier requires recruiting participants at earlier ages, but the ideal age range is currently unclear. To date, the most conventional method of recruitment is to enroll participants with a diagnosis of mild AD or mild cognitive impairment (MCI; for DMT) who are within a typically large age range. A systematic review of 165 RCTs for AD treatments found that the mean age of participants was 73.6 years (SD = 8.2),^12^ while 90% of RCTs for AD define a range from 55 to 85 years old, 20% of RCTs allow 50 to55 years old, and 15% allow for 85‐ to 90‐year‐old participants.

In order to identify the optimal age to intervene earlier in the lifespan, approaches capable of pinpointing the earliest inflection point in the transition from healthy to pathological aging are required. While fluid and biomarkers are used for participant identification in RCTs,^13^ tests of cognition should provide a complementary approach for monitoring disease progression^14^ and to improve precision of recruitment as they represent abilities used daily which impact an individual's functioning. Some have also argued that for a treatment to be called disease‐modifying, it should show a beneficial effect on cognition^15^; therefore, sensitive measures are required to evaluate treatments^16^, ^17^ at different stages of the disease process. The most commonly used methods employed in RCTs to date have been based on changes observed in the very late stages of the disorder. One approach to identify the earliest point of cognitive decline is to model the trajectory of change in cognition by age by repurposing statistical approaches used in pharmacology research, namely Emax (i.e., maximum effect) models that are commonly used in modeling dose‐response.^18^

Previous work applying Emax modeling in Down syndrome, which is considered a genetic variant of AD^19^ has identified the trajectory of cognitive decline in the preclinical and prodromal stages of AD. These findings have shown that changes in cognition can be detected as early as 20 years before the average age of diagnosis. We have demonstrated that such results can be used to assist with decisions on the optimal age to intervene and used to calculate sample size estimation for AD prevention trials.^14^ While these results have assisted trail design for the first RCT of a DMT to treat AD in Down syndrome,^20^ it is currently unclear whether this approach can be applied to people with sporadic AD, where there may be more heterogeneity in terms of age of onset and progression.

### 1.1 Objective

The aim of this study was to validate Emax modeling as a novel method to retrospectively identify the earliest age of change in cognition of people who would ultimately develop sporadic AD and compare these individuals to healthy controls without AD.

## 2 METHODS

### 2.1 Study design and setting

We used data from a prospective longitudinal cohort study of adults aged 65 years old and older from Montpellier, France.^21^ Baseline assessments were completed between 1999 and 2002 with follow‐up assessments designed to occur at 2‐year intervals. For the purposes of the present study, we analyzed data from participants’ inclusion and second timepoint (T2, 4‐year wave) assessments.

**RESEARCH IN CONTEXT**

1. **Systematic review**: The authors reviewed the literature using the Scopus database. There have been several studies that have examined methods to improve randomized control trial design for Alzheimer's disease (AD) therapies. However, there is a lack of research considering age of participants and examining trajectories of cognitive decline in sporadic AD.
2. **Interpretation**: These data provide validation for Emax modeling as a novel method to identify participants for clinical trials of disease‐modifying therapies in AD. This approach would be used to improve randomized control trials by determining optimal age bands to recruit participants.
3. **Future directions**: Future work could seek to use Emax modeling in different tests of cognition to determine tests which could be used as cognitive endpoints in clinical trials.

### 2.2 Participants

Our sample consisted of participants taken from a larger sample of 2259 individuals aged between 65 and 90 years who were selected by random sampling from the electoral rolls of the Montpellier district as part of the Enquête Sante ´ Psychologique—Risques, Incidence et Traitement (ESPRIT) study of late‐life neuropsychiatric disorders.^21^ Due to variability in follow‐up time between assessments (2.8 years to 4.45 years), we sampled 1493 participants who had T2 data which was at least 3 years to a maximum of 4 years from their baseline assessment in order to reduce the impact of time between assessments on performance.

Baseline prevalent cases of all‐cause dementia were excluded from further analysis in order to focus on new cases of AD during the study time. The remaining participants were categorized into two groups based on whether they were ever diagnosed with possible and probable AD while enrolled in the study (*n* = 101) or did not receive a diagnosis of any subtype of dementia (*n* = 1392, the healthy control group). We chose to analyze data from people who had a confirmed AD diagnosis during their enrolment in the study as this allowed us to work backward to identify when these individuals began to exhibit cognitive decline from their baseline assessment, with the certainty that they would eventually develop AD. Dementia diagnosis was based on the DSM‐IV criteria.^22^ We included a healthy control group to validate the results found in people with AD.

### 2.3 Ethics

The study protocol was approved by the Ethical Committee of the University–Hospital of Bicêtre (France) and written informed consent was obtained from each participant.

### 2.4 Measures

We measured verbal fluency, executive functioning, and global cognitive functioning using tests which are sensitive to early cognitive changes in MCI and preclinical studies. The Isaacs Set Test^23^ was used to measure verbal fluency, the Trail Making Test, Part B^24^ assessed frontal executive functioning and the Mini‐Mental State Examination (MMSE)^25^ was used as a global measure of cognitive functioning.

### 2.5 Statistical analysis

Following previous methods,^14^ we fitted Emax models to cognitive data to examine their trajectory of change by increasing age. To identify the earliest age‐bands of changes in performance for each test of cognition we explored dose‐response relationships between performance change over a 3‐ to 4‐year period with increasing “doses” of age. In the Emax models, we assumed a sigmoidal relationship between performance decline and age in years. This constrained a baseline level of cognitive stability, followed by a constant period of decline that eventually asymptotes. To explore change in performance on a yearly basis, mean proportional change between timepoints was calculated across participants in 5‐year smooth moving‐average baseline age bands, starting at age 65 and subsequently incrementing by 1 year. Age bands with fewer than four observations and individual change score outliers (>1.5 times the interquartile range of the static 5‐year age‐band or due to clinical anomalies, such as substantial improvement in performance in an older adult) were excluded. Emax curves were fitted to people with AD and healthy controls separately for each cognitive test to compare participants who would eventually go on to develop AD with healthy controls.

Raw performance changes from the age‐band at EC_1_ (1% of the maximum effect of age on performance) from the tests of cognition were used to estimate the required sample sizes to compare groups in a hypothetical RCT where a pharmacological treatment would reduce aging‐related decline by 35% or 75% compared to placebo over a 2‐year period. Cohen's *d* was used to show the effect sizes of these hypothetical group differences. Sample size calculations were performed in GPower 3.1, using independent samples *t*‐tests (*α *= 0.05) and 80% power. R version 4.1.3^26^ was used for all analyses.

## 3 RESULTS

As presented in Table 1, the AD sample consisted of predominantly females and participants were mostly educated to high school level (36%). The mean age at enrolment was approximately 75 years old and on average participants were in their late 70s at T2. They were diagnosed with AD in their early 80s and over a third of the sample were APOE ε4 carriers. The healthy control group comprised of nearly 60% females and like the dementia group, participants were primarily of high school level education. They were in their early seventies at baseline and mid‐seventies at T2. Approximately 18% of healthy controls were apolipoprotein E (APOE) ε4 carriers.

**TABLE 1:** Demographics of people with AD and healthy controls.

Parameter	People with AD	Healthy controls
*n*	101	1392
Mean age at baseline in years (SD)	74.95 (5.63)	72.56 (5.33)
Mean age at T2 in years (SD)	78.7 (5.65)	76.34 (5.32)
Sex (%)		
Male	33 (32.67)	571 (41.02)
Female	68 (67.32)	821 (58.98)
Education level (%)		
<High school	30 (29.70)	310 (22.27)
High school	36 (35.64)	581 (41.74)
College	10 (9.90)	136 (9.77)
University	25 (24.75)	365 (26.22)
Mean age of AD diagnosis in years (SD)	82.41 (5.56)	–
APOE ε4 carrier (%)^a^	36 (36.73)	242 (17.60)

^a^ Dementia group, *n* = 98; healthy controls, *n* = 1375.

### 3.1 Earliest age‐band of changes in cognition for people with AD and healthy controls

**FIGURE 1:**
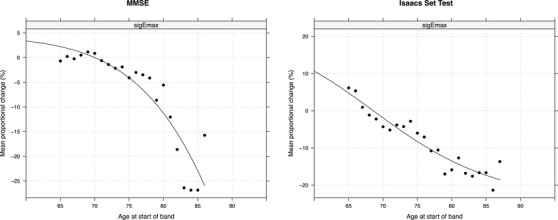
E_max_ curves for people with AD.

**FIGURE 2:**
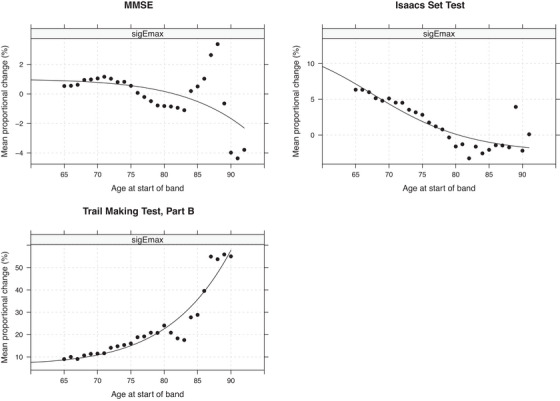
E_max_ curves for healthy controls.

For performance on both the MMSE and verbal fluency (Isaacs Set Test), the Emax curves for people with AD (Figure 1) showed a steeper and earlier decline compared to the healthy control group (Figure 2). Verbal fluency showed the earliest EC_1_ values in people with AD, demonstrating change in the 41–45 age‐band, and between the ages of 43 and 47 years for healthy controls (Table 2). This was followed by global cognitive functioning (MMSE) which showed the earliest decline from ages 67 to 71 years for people with AD, but much later in healthy controls with decline only starting in the 87–91 age band. The Emax model for executive functioning (Trail Making Test, Part B) failed to converge for people with AD. For healthy controls, it showed a late EC_1_ value, with the earliest changes in performance being detected between the ages of 85 and 89 years.

**TABLE 2:** Age‐bands of 1%, 5%, and 10% of the maximum effect of age on performance for people with AD and healthy controls

Parameter	Age‐band at EC_1_		Age‐band at EC_5_		Age‐band at EC_10_	
Outcome	Dementia group	Healthy controls	Dementia group	Healthy controls	Dementia group	Healthy controls
MMSE	67–71	87–91	79–83	103–107	85–89	111–115
Isaacs Set Test	41–45	43–47	50–54	51–55	54–58	55–59
Trail Making Test, Part B	–	85–89	–	101–104	–	108–112

Abbreviation: MMSE, Mini‐Mental State Examination.

**TABLE 3:** Sample size estimations for people with AD for the EC_1_ age‐bands

Parameter				Treatment at 35% efficacy			Treatment at 75% efficacy		
Outcome	Age‐band at EC_1_	Mean expected change in control group	Pooled SD	Mean expected change in treatment group	Effect size (*d*)	Total required sample size	Mean expected change in treatment group	Effect size (*d*)	Total required sample size
Isaacs Set Test	77–81^a^	–4.13	8.03	–2.68	–0.18	768	–1.03	–0.39	168
MMSE	67–71	–0.05	1.15	–0.03	–0.02	106,042	–0.01	–0.03	23,096

^a^ Not EC_1_ value. Calculated using independent samples one‐sided *t*‐tests (α = 0.05) and 80% power.

Abbreviation: MMSE, Mini‐Mental State Examination.

### 3.2 Sample size estimations for a hypothetical RCT people with AD

The EC_1_ age‐band for verbal fluency was 41–45 years old, which is substantially younger than our data would allow to calculate a sample size for a hypothetical RCT. However, as Table 3 demonstrates, the 77–81 age‐band showed an achievable sample size for an RCT with a total sample size of 768 (Cohen's d = −0.18) with a treatment which was 35% effective and this test as the cognitive endpoint. With an AD treatment which was 75% effective, an RCT would require total of 168 participants (Cohen's d = −0.39). Using the EC_1_ age‐band of 67–71 years old for global cognitive functioning, an RCT with a treatment which was 35% effective would require a total of 106,042 participants (Cohen's d = −0.03) and 23,096 (Cohen's d = −0.03) if the treatment was 75% effective.

## 4 DISCUSSION

We found that in participants who ultimately develop AD, the earliest changes in general cognitive functioning and verbal fluency can be detected considerably earlier than the mean age of AD diagnosis in our sample (mean = 82 years old, SD = 5.56). Moreover, mean age of AD diagnosis was similar to AD onset previously reported in the literature (80 years old).^27^ Importantly, we demonstrated a later trajectory of change in the large healthy population of older adults who did not develop AD.

Previous authors have suggested one of the reasons RCTs in AD have been largely unsuccessful is because of poor trial design.^28^ There has been substantial work on methods to improve RCT design including research on participants’ priorities to increase willingness to participate in RCTs,^29^ suggestions to use Bayesian adaptive RCT designs,^30^ and using polygenic hazard scores to stratify participants.^31^ However, limited consideration has been given to the age of participants when they enter into an RCT and the accepted convention is to enroll participants over 50, 60, or 65 years to 85 years old depending on the target population, study aims or choice of outcome measure. Age is the most salient risk factors for AD, with the possibility of a diagnosis doubling every 5 years after age 60 years.^32^ By 85 years and older, a diagnosis of AD is nearly 14 times higher in this age group compared to people aged 65–69 years old.^33^ Yet these individuals are all collapsed in a single group for RCTs. We used age of participants in age‐bands to model how they performed on measures of cognition with increasing age. Our results suggest that Emax modeling could contribute and benefit RCT development by guiding the optimal recruitment period to maximize any potential benefits of treatments which could prevent or delay the development of AD.

An important consideration for an effective treatment is to ensure that it is administered to the right participants at the right time. RCTs which are more selective in the age range of participants and recruit participants when they exhibit the earliest signs of decline could potentially improve the success of DMT by intervening earlier in the disease stage. This approach could also help to provide better data related to treatment efficacy,^30^ which is currently lacking in the literature. Several authors have made recommendations for earlier detection to be utilized in RCTs to optimize intervention windows.^11^, ^34^ These data provide validation of Emax modeling as a viable approach to identify the earliest inflection of change in cognition to intervene earlier in the AD disease course.

As previously shown, combining trajectory modeling with sample size calculations is important to identify appropriate measures which can be used as cognitive endpoints in RCTs.^14^ The results from the present study emphasize this consideration. While the measure of global cognitive functioning (MMSE) was able to detect the earliest changes in cognition up to 15 years before the mean age of AD diagnosis within our sample, the decline in performance was negligible and it would require a sample of 23,096 participants. The MMSE is commonly used in RCTs and was used as a cognitive endpoint in studies of DMT such as aducanumab,^35^ but our results suggest that while it may be sensitive to early changes, the sample size calculations demonstrated that it may not be particularly useful as a cognitive endpoint due to the large numbers needed to detect a change in performance between control and treatment groups.

The results from the verbal fluency task suggest that this cognitive ability shows the earliest decline in the 4th decade of life in people who eventually develop AD. Tests of verbal fluency and visuo‐spatial memory are known to be sensitive to early AD related changes with decline trajectories being evident in the decade preceding diagnosis.^36^ Moreover, our previous work with Emax modeling and verbal fluency has demonstrated that it is sensitive to the earliest AD‐related change in adults with Down syndrome.^14^ Another study in adults with Down syndrome showed that verbal fluency performance was negatively correlated with the established AD biomarkers, neurofilament light, and glial fibrillary acidic protein.^37^ However, the EC_1_ age‐band (1% of the maximum effect of age on performance) for the healthy control group was also a similar age band (43–47 years old) as the AD group (41–45 years old). Verbal fluency is a test which is highly sensitive to changes in multiple brain regions^38^, ^39^; therefore, the early changes and early age bands identified could reflect general age‐related changes instead of AD related changes in these participants. These results should be examined further and future work using Emax modeling could investigate the trajectory of change using a lifespan approach by incorporating participants across a broader age range including those in midlife and/or younger to examine the robustness of the current findings.

### 4.1 Strengths and limitations

Utilizing data from people who ultimately developed AD, we have shown that there is benefit to refining the age of recruitment in identifying potential participants, since trajectory modelling can pinpoint the earliest decline in cognitive abilities, even in people with sporadic AD, which is considerably more heterogenous^40^ than genetic variants which exhibit a more predictable onset.^41^ We included only participants who had an AD diagnosis and excluded participants with other dementias making our findings more applicable to current RCTs which are primarily conducted in people with AD. Moreover, our healthy controls and AD participants were sampled from community dwelling adults increasing the generalizability of their performance on the measures of cognition. Our results also provide replication and validation data for previous work in Down syndrome^14^ but in a larger sample size from the general population. Finally, we utilized a novel and innovative approach to address a neglected aspect of RCT design which could improve treatment outcomes.

There are several limitations which should be addressed. First, the cognitive tests used in the present study may not be the preeminent choice as cognitive endpoints for an AD prevention RCT, and more sensitive tests could be examined in future work. We also did not include measures of functional abilities which are important to consider as outcome in RCTs.^42^ AD is a heterogeneous condition^43^ and performance on cognitive tests can vary. Moreover, our sample size was limited to 101 AD participants. While we made efforts to account for this heterogeneity by calculating performance in 5‐year smooth moving‐average baseline age bands, the variability in performance found in AD could have impacted our results. Furthermore, we only considered age as a risk factor, but there are many other variables that can increase a person's risk of AD including APOE.^44^ Future studies would benefit from considering the impact of these variables using a larger sample within the age ranges exhibiting the earliest changes in cognition to understand how these may contribute to risk of cognitive decline due to AD.

## 5 CONCLUSION

These results suggest that trajectory modeling could be used to enrich and improve RCT design by providing data on the ideal age range to intervene. We have shown that sensitivity to change depends on the primary outcome as well as the age of participants, so authors designing RCTs need to ensure the population and primary outcome are consistent with the research question. Combining this approach with markers of risk such as APOE e4 or those with biomarker evidence of potential early AD (e.g., amyloid PET) could help to design more efficient RCTs, particularly for those targeting the stages of disease before an AD diagnosis.

## CONFLICT OF INTEREST STATEMENT

André Strydom received consulting fees from AC Immune and Alnylam and is on an Advisory Board for AC Immune. All other authors declare no conflicts of interest. Author disclosures are available in the supporting information.

## CONSENT STATEMENT

All human subjects provided written informed consent.

## Supporting information

Supporting Information
